# How Do You #relax When You’re #stressed? A Content Analysis and Infodemiology Study of Stress-Related Tweets

**DOI:** 10.2196/publichealth.5939

**Published:** 2017-06-13

**Authors:** Son Doan, Amanda Ritchart, Nicholas Perry, Juan D Chaparro, Mike Conway

**Affiliations:** ^1^ Deparment of Biomedical Informatics University of California, San Diego La Jolla, CA United States; ^2^ Linguistics Department University of California, San Diego La Jolla, CA United States; ^3^ Department of Psychology University of Utah Salt Lake City, UT United States; ^4^ Department of Biomedical Informatics University of Utah Salt Lake City, UT United States

**Keywords:** social media, Twitter, stress, relaxation, natural language processing, machine learning

## Abstract

**Background:**

Stress is a contributing factor to many major health problems in the United States, such as heart disease, depression, and autoimmune diseases. Relaxation is often recommended in mental health treatment as a frontline strategy to reduce stress, thereby improving health conditions. Twitter is a microblog platform that allows users to post their own personal messages (tweets), including their expressions about feelings and actions related to stress and stress management (eg, relaxing). While Twitter is increasingly used as a source of data for understanding mental health from a population perspective, the specific issue of stress—as manifested on Twitter—has not yet been the focus of any systematic study.

**Objective:**

The objective of our study was to understand how people express their feelings of stress and relaxation through Twitter messages. In addition, we aimed at investigating automated natural language processing methods to (1) classify stress versus nonstress and relaxation versus nonrelaxation tweets, and (2) identify first-hand experience—that is, who is the experiencer—in stress and relaxation tweets.

**Methods:**

We first performed a qualitative content analysis of 1326 and 781 tweets containing the keywords “stress” and “relax,” respectively. We then investigated the use of machine learning algorithms—in particular naive Bayes and support vector machines—to automatically classify tweets as stress versus nonstress and relaxation versus nonrelaxation. Finally, we applied these classifiers to sample datasets drawn from 4 cities in the United States (Los Angeles, New York, San Diego, and San Francisco) obtained from Twitter’s streaming application programming interface, with the goal of evaluating the extent of any correlation between our automatic classification of tweets and results from public stress surveys.

**Results:**

Content analysis showed that the most frequent topic of stress tweets was education, followed by work and social relationships. The most frequent topic of relaxation tweets was rest & vacation, followed by nature and water. When we applied the classifiers to the cities dataset, the proportion of stress tweets in New York and San Diego was substantially higher than that in Los Angeles and San Francisco. In addition, we found that characteristic expressions of stress and relaxation varied for each city based on its geolocation.

**Conclusions:**

This content analysis and infodemiology study revealed that Twitter, when used in conjunction with natural language processing techniques, is a useful data source for understanding stress and stress management strategies, and can potentially supplement infrequently collected survey-based stress data.

## Introduction

Psychological stress has been linked to multiple health conditions, including depression [1], heart disease [2], autoimmune disease [3], and general all-cause mortality [4]. Stress has also been associated with worse health outcomes among those living with chronic illness [5], suggesting that stress may exacerbate preexisting health conditions, as well as contribute to the development of new health problems. Stress not only contributes to physical and mental health problems, such as heart disease, depression, and autoimmune diseases [6], but also has negative impacts on family life and work, significantly impairing quality of life [7,8]. Accordingly, stress is an important concern for public health prevention initiatives [7,8].

Health surveys have demonstrated that stress negatively affects a large proportion of the US population [9]. Underscoring the magnitude of the problem, a study conducted by the Harvard School of Public Health found that 49% of the American public reported being stressed within the last year, and also found that 60% of those who reported being in poor health also reported experiencing a substantial amount of stress within the last month [7]. Further, levels of stress appear to be unequally distributed throughout the population [10]. National surveys have documented that higher levels of stress are reported among those who have lower income, are less educated, and are younger [11]. Theorists have suggested that geographic clustering of psychological characteristics may be driven by selective migration (in this case, people more vulnerable to stress seek out others like themselves), social influence (ie, people with attitudes and beliefs that lead to greater stress cluster together geographically), or environmental influence (ie, features of the physical environment, such as neighborhoods, increase stress among those who live close to one another) [12]. In short, large-scale studies have documented both the high prevalence of stress within the United States and geographic clustering of psychological distress, suggesting that symptoms of stress should ideally be tracked at both the national and local levels.

Relaxation is considered a key component of frontline stress management techniques, such as cognitive-behavioral stress management [13]. General stress management can include adaptive coping (eg, distraction), physical relaxation strategies (eg, diaphragmatic breathing), cognitive reappraisal (eg, reconsidering the stressor from a different perspective), and mindfulness (ie, increasing awareness of the present moment). These stress management strategies are intended to reduce psychological and physiological arousal related to stress, promote healthier coping alternatives, and, in turn, reduce some of the negative health impacts of stress. Indeed, these strategies have been found to be effective for improving health outcomes among those living with chronic illness [14-16], as well as for improving general mental health and quality of life [17,18].

Understanding what the major causes of stress are and how people negatively or positively manage their stress (eg, through stress management techniques such as cultivating relaxation) is important [7,19]. Population health surveys often use telephone interviews or questionnaires from samples of the population, such as the US Centers for Disease Control and Prevention’s (CDC) Behavioral Risk Factor Surveillance System [20]. These methods, although reliable, are conducted relatively infrequently due to cost and may be less effective at reaching certain populations, such as those without a dedicated landline telephone. With the rapid growth of online social networks today, social media data can serve as a useful additional resource to understand aspects of stress that are difficult to assess in general surveys or clinical care. For example, social media provide a means to rapidly and dynamically address new and evolving research questions with a degree of flexibility not possible with surveys. Social media may also provide insights into populations that may be underrepresented in surveys (depending on the demographics of the particular social media platform used). Thus, social media can potentially serve as a beneficial supplement to detailed surveys when trying to understand public health concerns.

Twitter—one of the most popular social media platforms—is a microblog service that allows users to post their own personal messages (a “tweet” with a 140-character limit). As of May 2016, it had 310 million active users with 1 billion unique visits monthly to sites with embedded tweets [21]. The utility of Twitter as a data source has been investigated in numerous applications such as election prediction [22], stock market prediction [23], oil price changes [22], and earthquake and disasters [24].

Twitter has also been used in public health for tracking influenza [25-27], and for studying breast cancer prevention [28], childhood obesity [29], issues related to general health [30], tobacco and e-cigarette use [31], dental pain [32,33], general pain [34], sexually transmitted diseases [35], and weight loss [36]. There has also been research regarding the general well-being of people in different geographical locations using Twitter messages [37], and correlation studies of Twitter messages with depression [38] and with heart disease mortality [39]. However, to our knowledge, no studies specifically focused on stress and stress management have been conducted until now.

In this study, we investigated how people express their own stress and relaxation through an in-depth content analysis of Twitter messages. In addition, we investigated automated methods to classify stress and relaxation tweets using machine learning techniques. Furthermore, we ranked stress and relaxation levels based on the relative proportions of stress- and relaxation-related tweets (as identified by our natural language processing classifiers) originating in 4 US cities: New York, Los Angeles, San Diego, and San Francisco. We then compared these results with public surveys reported by Forbes and CNN [40,41]. Using easily acquired, naturalistic Twitter data, and complementing existing survey-based epidemiological methods, this study provides another perspective on how people think about and cope with stress.

## Methods

### Data Collection

#### Dataset 1

To begin our investigation of stress and relaxation (stress management) tweets, we first collected tweets with user-defined stress and relaxation topics using the Twitter REST application programming interface (API) [42]. The user-defined topics included the hashtagged topics #stress and #relax, as well as variations of these words. Textbox 1 lists the full search list we used. We collected tweets between July 9 and July 14, 2014. We supplemented this seed dataset with tweets from the random sample stream Twitter streaming API [43] (1% sample rate) in order to have better representation of “everyday” tweets that did not necessarily contain stress- and relaxation-related hashtags, but that still contained the keywords “stress” or “relax.” This dataset consisted of 1326 stress-related and 781 relaxation-related tweets. We referred to this dataset as dataset 1.

**List of hashtags related to stress and relaxation to create dataset 1.**

**Stress-related hashtags**

#stress

#stressed

#stressful

#stressin

#stressing

#sostressful

#sostressed

#stressinout

#stressingout

**Relaxation-related hashtags**

#relaxed

#relaxin

#relaxing

#sorelaxin

#sorelaxing

#### Dataset 2

We further investigated the characteristics of stress and stress management by geographical location (4 US cities) and compared the locations against each other using dataset 2. This dataset—much larger than dataset 1—consisted of geotagged tweets obtained from the Twitter streaming API [43] in 1 of 4 possible cities: Los Angeles, New York, San Diego, and San Francisco. We chose these cities because they are densely populated and major metropolitan areas on the east and west coasts of the United States. Tweets were collected between September 30, 2013 and February 10, 2014. The number of tweets for each city for this time period was 8.2 million for New York, 6.6 million for Los Angeles, 3 million for San Diego, and 4.4 million for San Francisco. Note that the most populous cities—that is, New York and Los Angeles—generated the greatest number of tweets during the study period. We referred to this dataset as dataset 2.

### Criterion Standard and Manual Analysis of Tweets

Since our primary goal in this study was to understand how people express stress and relaxation through Twitter, we developed annotation guidelines for both stress and relaxation tweets based on reports from the American Psychological Association [7], CDC [8,44], and medical websites [6,45,46]. Following these guidelines, we classified tweets by both genre and theme. Genre reflects the format of the tweet (eg, personal experience), and theme reflects the domain of the actual content conveyed (including such categories as stress symptoms and stress topics).

Details for each genre and theme for stress and relaxation tweets were as follows.

#### Genre

We categorized tweets as being first-hand experience versus other genres. We defined first-hand experience as a direct personal experience, or an experience directly related to the user writing the tweet. Other genres were second-hand experience, advertisements, news articles, etc. This genre classification was based on previous work on classifying health-related tweets [31]. After classifying a tweet as first-hand experience, we assigned its content into 2 themes: stress and relaxation.

#### Stress Themes

Content analysis focused on 3 main questions: (1) What kind of stress was being experienced? (2) What was the cause of the stress? and (3) What kind of actions, if any, were being taken regarding the stress? Based on these questions, we categorized the theme into 3 categories: stress symptoms, topics, and action(s) taken.

Symptoms fell into 3 classes: (1) psychological and emotional, (2) physical, and (3) behavioral. These categories were based on guidelines for stress symptoms [47-49].

Topics referred to the general topic of a tweet: (1) work, (2) education, (3) finances, (4) social relationships, (5) travel, (6) temporal, and (7) other. These topics were identified based on an analysis of data from dataset 1.

The action taken theme indicated the action that people reported taking when they were stressed. The action could be either negative or positive. An example of a negative action is “I need a drink tonight. #sostressed.” An example of a positive action is “I need a nap, and a hug. #stressingout #tired.”

The nonspecific theme was for users who simply tweeted without any symptom, topic, or action; for example, “#stressed!!!,” “Bad Night :,(” and “#SoStressed.”

#### Relaxation Themes

We categorized first-hand experience relaxation tweets by the following topics (themes), which referred to the action reported being taken by the user to relax, such as exercising or listening to music. We created 11 topics based on data from dataset 1: (1) physical, (2) water, (3) self-care, (4) alcohol & drugs, (5) entertainment & hobbies, (6) food & drink, (7) nature, (8) rest & vacation, (9) social relationships, (10) other, and (11) nonspecific.

**Figure 1:**
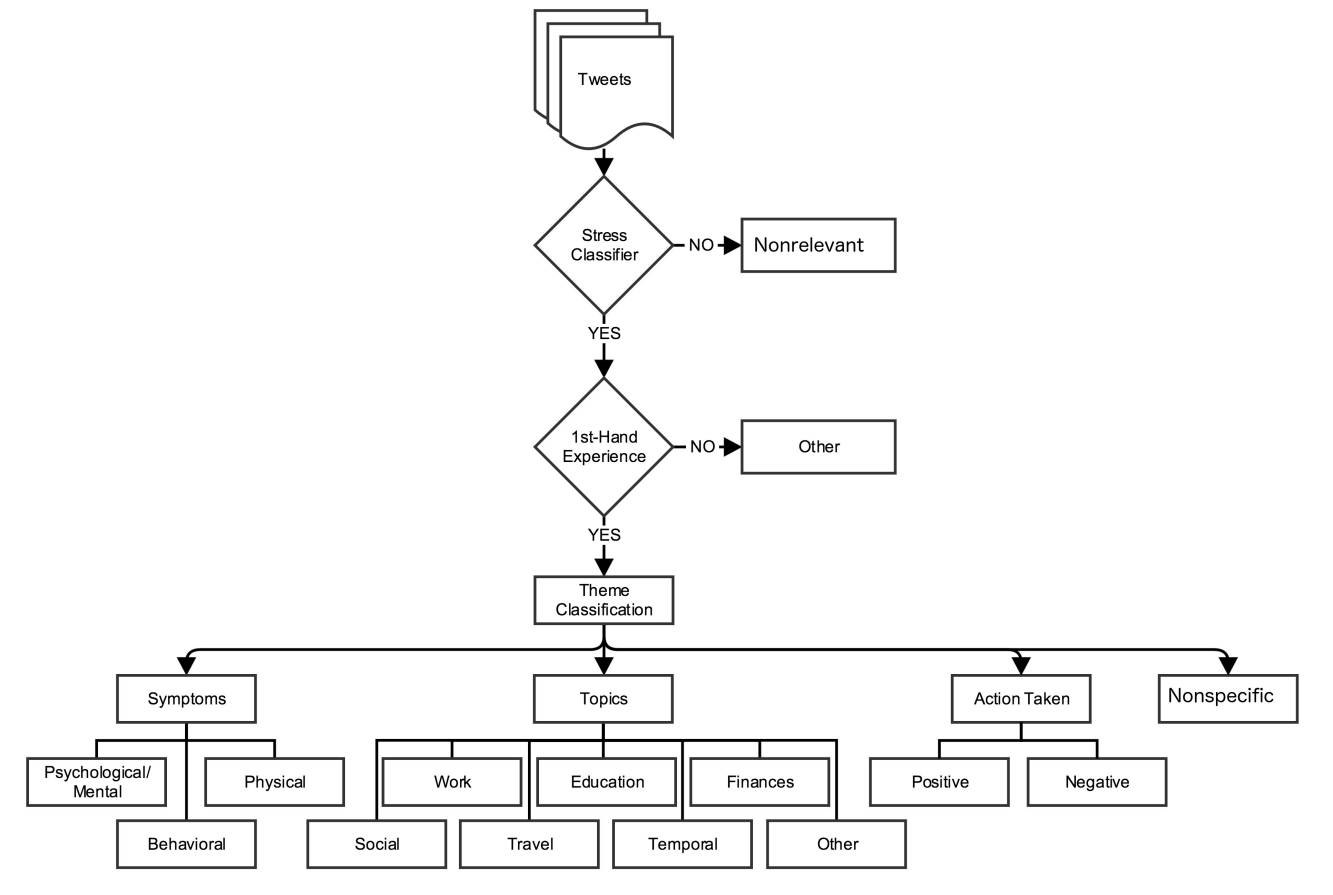
Schema used to classify stress tweets.

**Figure 2:**
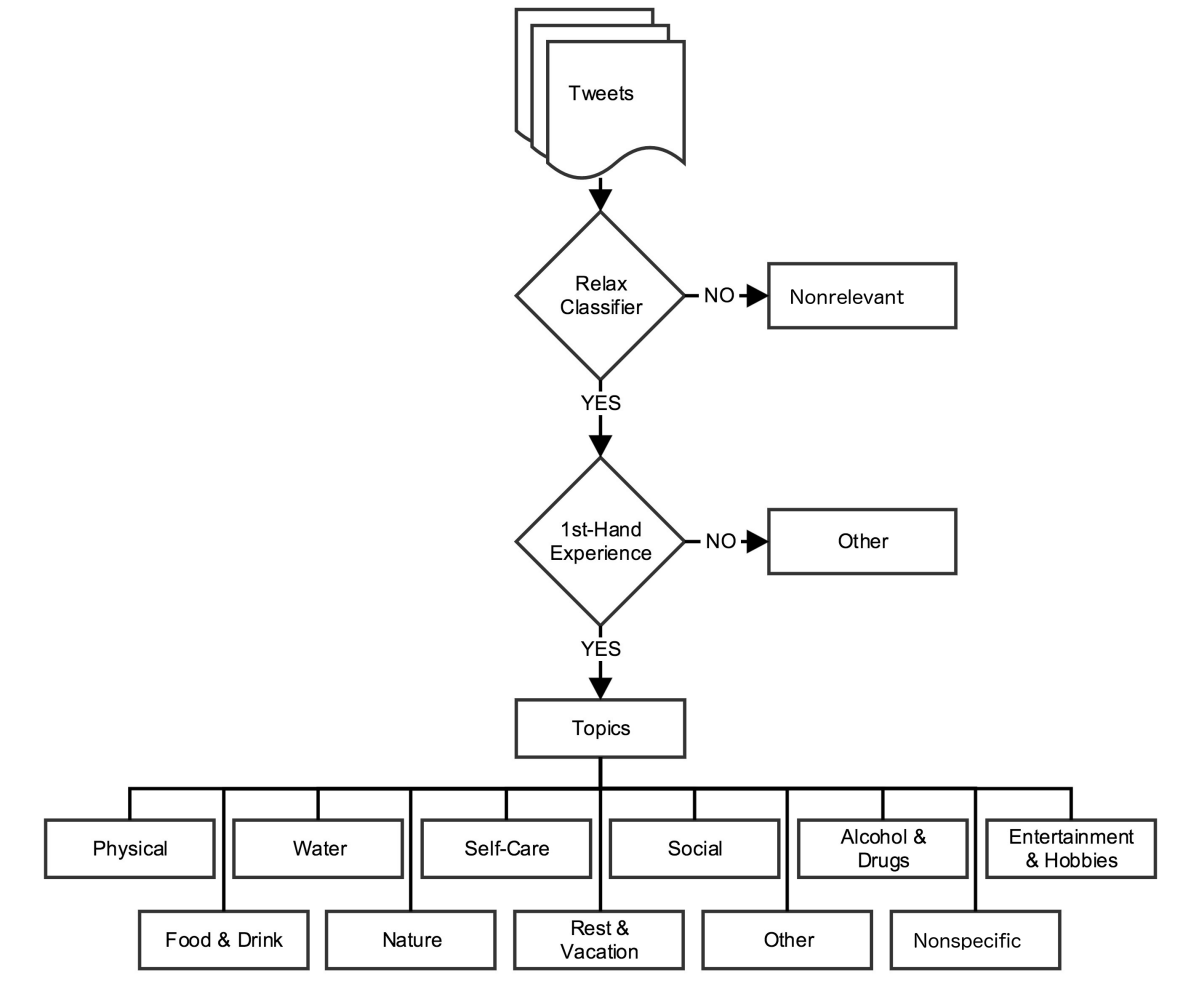
Schema used to classify relaxation tweets.

Figure 1 depicts the schema for stress tweets and Figure 2 depicts the schema for relaxation tweets. Definitions and examples of each category of first-hand experience tweets and its themes for stress and relaxation tweets are listed in Multimedia Appendix 1 and Multimedia Appendix 2, respectively.

**Figure 3:**
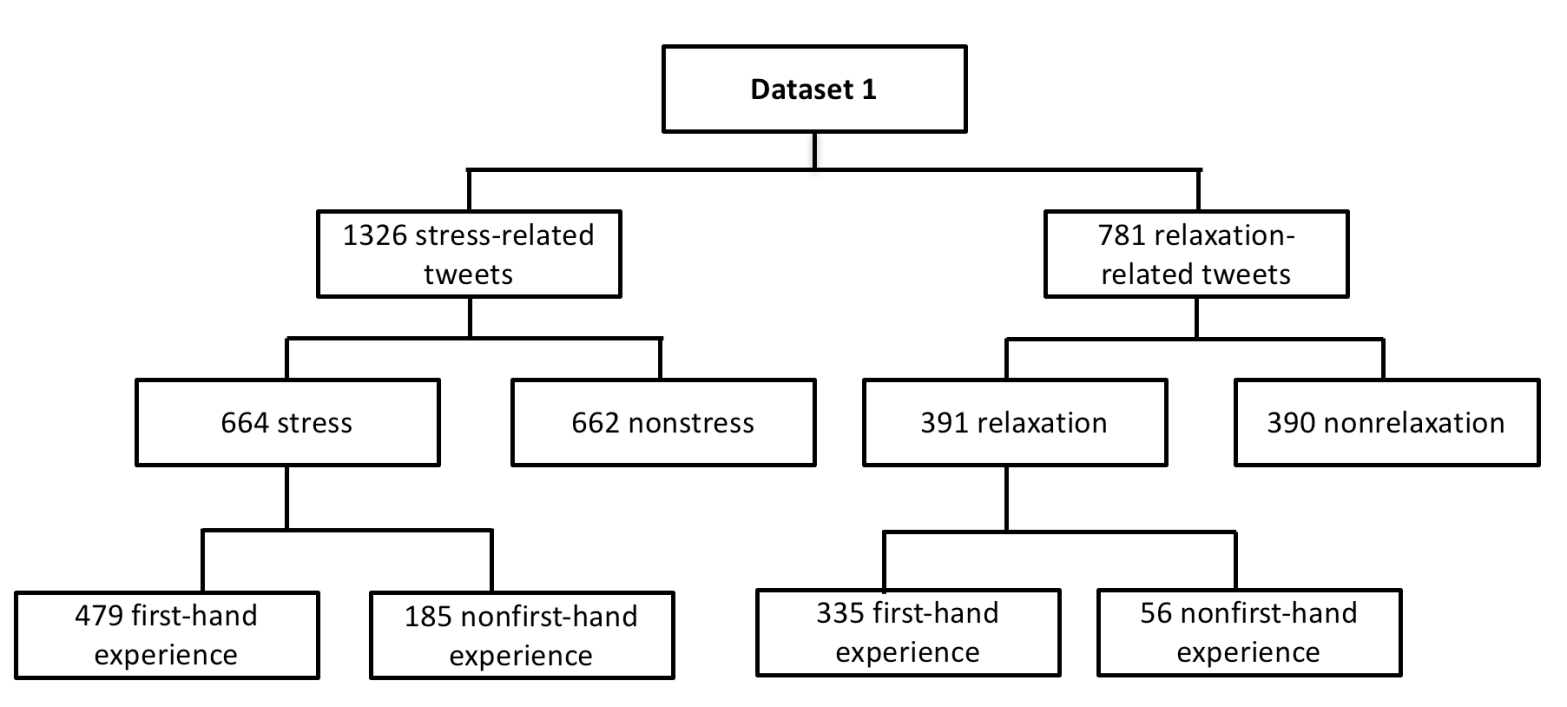
Description of dataset 1.

One author (AR) annotated stress and relaxation tweets from dataset 1 and another (SD) annotated and verified the dataset to ensure that all tweets were annotated correctly. Any disagreements were resolved by meetings or exchanging emails. Dataset 1 contained a total of 664 stress and 662 nonstress tweets among the 1326 stress-related tweets, and a total of 391 relaxation and 390 nonrelaxation tweets among the 781 relaxation-related tweets. For each stress or relaxation tweet, 2 authors (AR, SD) discussed and manually annotated tweets based on the guidelines as described above. After annotation, there were a total of 479 stress tweets and 335 relaxation tweets related to first-hand experience in dataset 1. Figure 3 depicts the details of dataset 1.

Since the prevalences of some of the stress themes (eg, finances, work) and relaxation themes (eg, food & drink, social) in dataset 1 were very low (ie, too infrequent to train a machine learning classifier), we developed an automatic keyword-based theme classifier using a manually crafted lexicon of stress and relaxation keywords associated with each category. We first generated unigrams and bigrams from dataset 1, and one author (AR) manually reviewed and selected the highest-frequency unigram and bigram keywords. We then manually added corresponding synonyms into each theme to increase the coverage of the classifier. For example, the topic “education” in the stress schema contained the unigrams “school,” “college,” and “classes” and the bigram “high school” in dataset 1. We manually added synonyms of those terms, such as “exams” and “studying” as unigram keywords and “college life,” “my tuition,” and “on finals” into bigram keywords. The list was iteratively reviewed and confirmed by another author (SD). There was an average of 20 unigram and 20 bigram terms for each theme. We created only unigram and bigram keywords, since tweet messages are short in nature. Bigram keywords were necessary to include idiomatic expressions like “vicious cycle” and “hate feeling,” and they also added more specificity, such as “my heart” and “my sanity,” which helped to increase the accuracy of the classifiers.

### Machine Learning Algorithms

**Figure 4:**
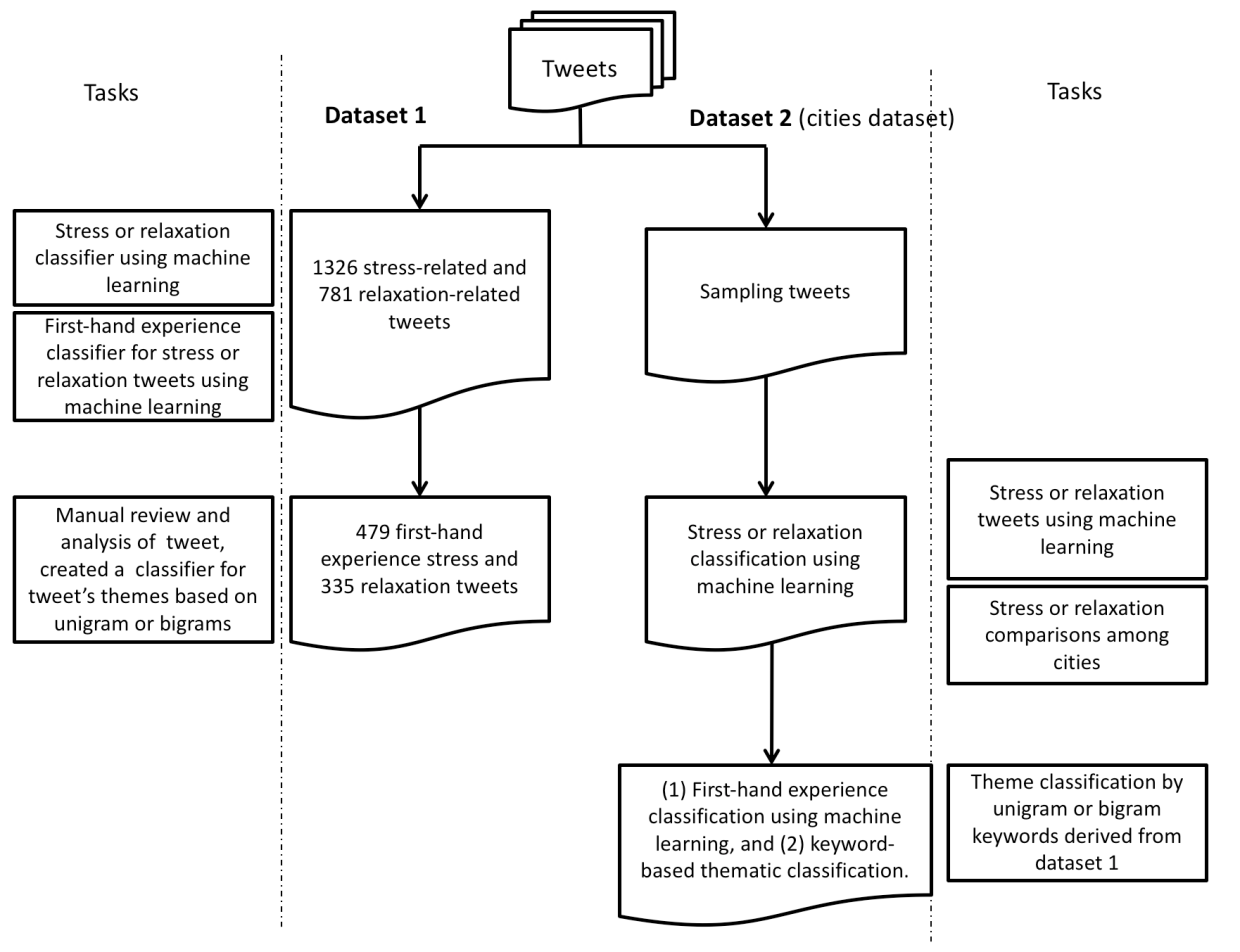
Datasets and tasks used for machine learning.

Leveraging the annotated data derived from our content analysis of dataset 1, we applied and evaluated machine learning algorithms for classification of stress versus nonstress tweets and relaxation versus nonrelaxation tweets (on dataset 1). To apply the classifier trained on dataset 1 to the unseen, much larger dataset 2 (cities dataset), we first filtered tweets by keeping only the tweets that contained stress- or relaxation-related hashtags in Textbox 1 or the keywords “stress” or “relax” for each city in dataset 2. After this step, dataset 2 contained only tweets with stress- or relaxation-related keywords or hashtags. To calculate the proportion of stress or relaxation tweets at the city level, we used the stress or relaxation classifier trained on dataset 1 to filter stress or relaxation tweets, and then applied the classifier for first-hand experiencer to tweets from each city in dataset 2. Figure 4 shows a flowchart describing our machine learning design.

Our study focused on 2 machine learning-based classification tasks. First, tweets were classified into the appropriate stress and relaxation category (ie, is it stress or relaxation related?). Second, first-hand experience tweets versus nonfirst-hand experience tweets were classified. We used 2 machine learning algorithms: naive Bayes and support vector machines (SVMs), which were implemented on dataset 1 using 10-fold cross-validation. We used both the naive Bayes and SVM algorithms, as both these algorithms have been used extensively for text classification tasks [50-52]. We used the *Rainbow* package [51] for implementing both naive Bayes and SVMs (linear kernel). We used “bag-of-words” as feature sets for both algorithms. The reason we used the bag-of-word representation is that this feature representation is considered as a baseline and the most common text representation in text classification in general [50-52]. To the best of our knowledge, this is the first study on classifying tweets on stress and relaxation tweets.

### Calculating the Proportion of Stress and Relaxation Tweets at the City Level

We applied the 2-step classification to each city in dataset 2 to automatically identify stress and relaxation tweets. We calculated the proportions of stress and relaxation tweets to the total number of tweets in each city.

### Measurements and Statistical Analysis

For both stress or relaxation and first-hand experience classifications, we used accuracy, sensitivity, specificity, and positive predictive values (PPVs) as metrics [53-55]. They were defined as follows: sensitivity = TP/(TP + FN); PPV = TP/(TP + FP); specificity = TN/(FP + TN); and accuracy = (TP + TN)/(TP +TN + FP + FN), where TP is the number of tweets that are correctly classified as true, FP is the number of tweets that are incorrectly classified as true, FN is the number of tweets that are true but incorrectly classified as false, and TN is the number of tweets that are correctly classified as false.

To compare data among cities, we used Pearson chi-square test and reported significance if the *P* value was less than .05 [56]. Statistical analyses were performed using the publicly available R package software version 3.2.3 (R Foundation). Note that, to preserve the anonymity of Twitter users, all example tweets reported in this paper are paraphrases of original tweets.

## Results

### Content Analysis in Stress and Relaxation Tweets (Dataset 1)

**Figure 5:**
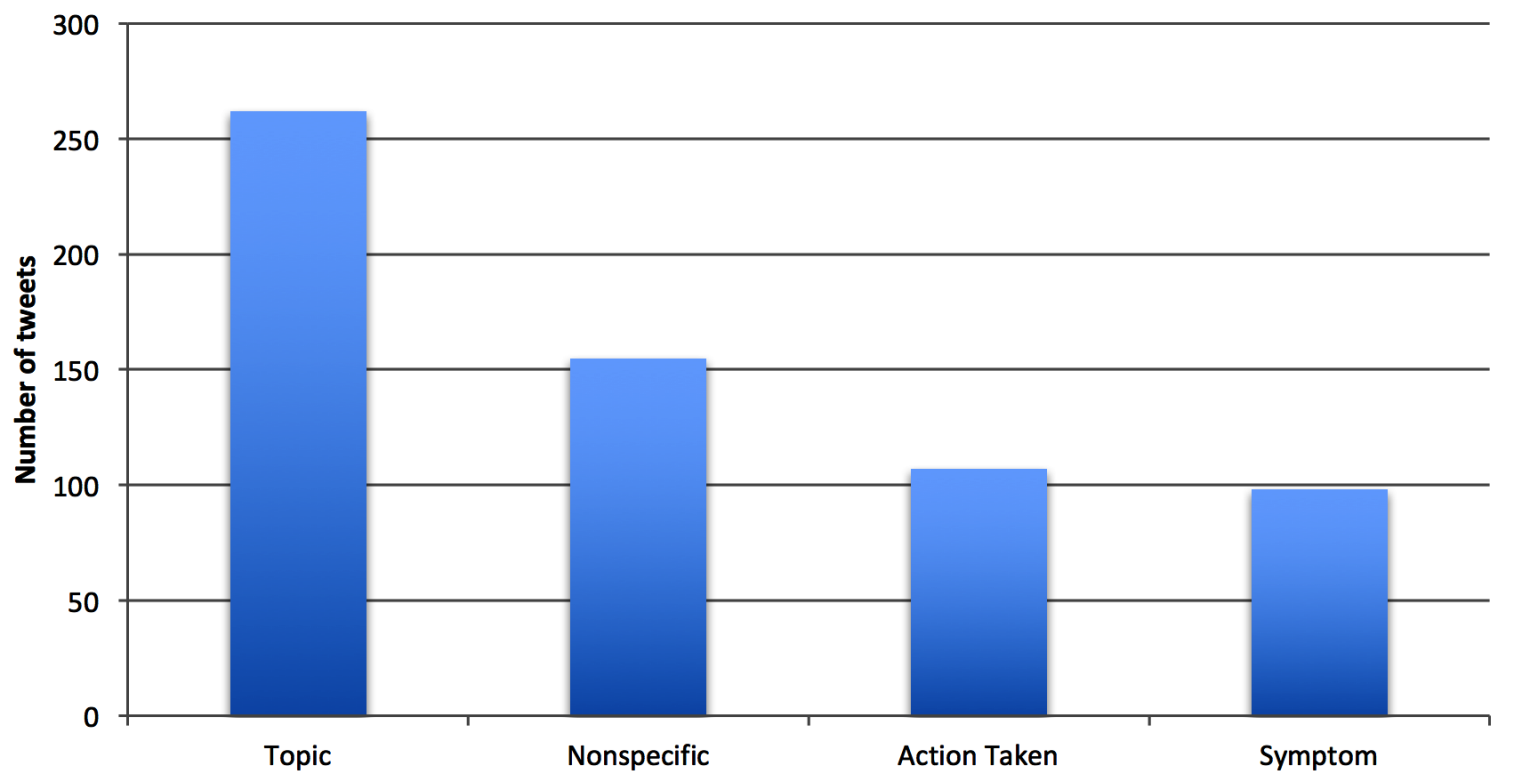
Distribution by theme of first-hand experience stress tweets in dataset 1.

Figure 5 shows the distribution of themes in first-hand experience stress tweets. The highest-frequency theme in stress tweets was topic, followed by nonspecific (eg, “#stressed!!!”), action taken (eg, “I need a drink #sostressed”), and symptoms (eg, “Not sure what to do...#stressed #worried #lost”). This suggests that Twitter users who posted about stress usually posted more about the cause or topic of their stress and less about actions and symptoms associated with stress.

**Figure 6:**
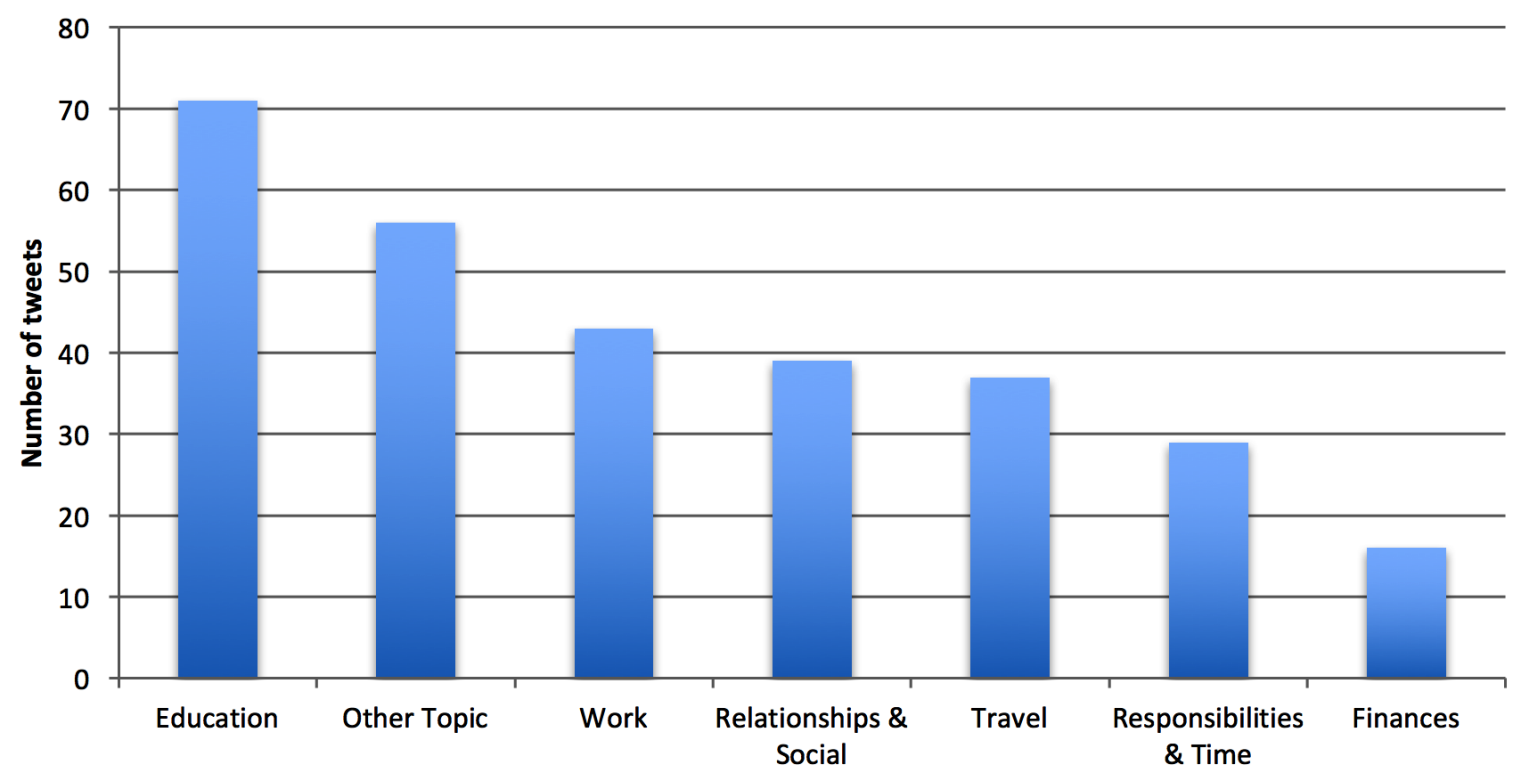
Distribution by topic of first-hand experience stress tweets in dataset 1.

Among the total number of stress-related tweets, as Figure 5 shows, the most frequent topic was education, followed by other topic, work, and social relationships. This is interesting because many of Twitter’s users are young people who attend school [57,58]. It seems that education and issues related to education, such as exams and finals, were of the utmost concern for Twitter users. Examples of the education topic are “Never doing a session B math course ever again #sostressful” and “my exam in less than a month?! #stressing.” Figure 6 shows the topic distribution of first-hand experience stress tweets.

**Figure 7:**
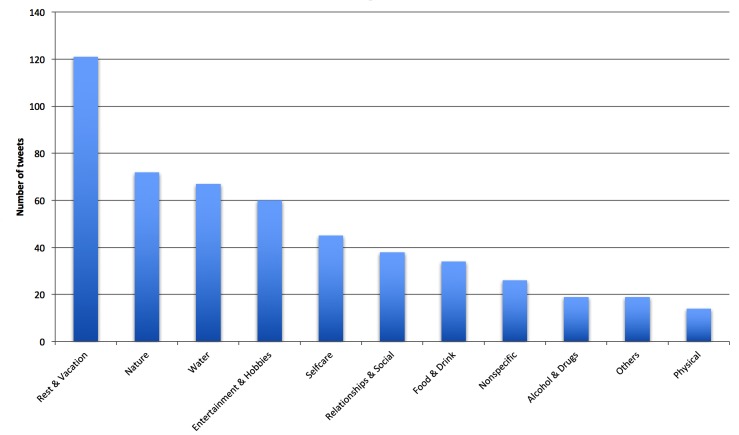
Distribution by topic of first-hand experience relaxation tweets in dataset 1.

Relaxation-related tweets encompassed a wider range of topics than stress-related tweets. The most frequent topic of relaxation tweets was rest & vacation, followed by nature and water. Figure 7 shows topic distribution of first-hand experience of relaxation tweets.

### Automatic Classification of Stress and Relaxation Tweets (Dataset 1)

Table 1 shows cross-validated classification results. Our results indicated that both algorithms achieved high accuracy (range 78.08%-85.64%), sensitivity (range 90.26%-99.09%), and PPV (range 70.68%-89.32%). Specificity was rather lower, especially with first-hand relaxation classification (naive Bayes: 11.67%, SVM: 18.33%).

**Table 1:** Classification evaluation using 10-fold cross-validation on dataset 1.

Classification	Machine learning algorithm							
	Naive Bayes				Support vector machine (linear kernel)			
	Acc^a^ (%)	Sen^b^ (%)	Spec^c^ (%)	PPV^d^ (%)	Acc (%)	Sen (%)	Spec (%)	PPV (%)
Stress vs nonstress	78.64	91.97	65.30	72.69	81.66	92.73	70.61	76.07
Relaxation vs nonrelaxation	78.08	96.15	60.00	70.68	83.72	90.26	77.18	79.86
First-hand vs nonfirst-hand experience stress	87.58	95.53	67.89	88.14	85.61	90.64	73.16	89.32
First-hand vs nonfirst-hand experience relaxation	85.64	99.09	11.67	86.07	83.85	95.76	18.33	86.56

^a^Acc: accuracy.

^b^Sen: sensitivity.

^c^Spec: specificity.

^d^PPV: positive predictive value.

Of the 2 machine learning algorithms used, SVM (with linear kernel) performed better than naive Bayes in classifying stress versus nonstress tweets (81.66% vs 78.64% accuracy, 92.73% vs 91.97% sensitivity, 70.61% vs 65.30% specificity, 76.07% vs 72.69% PPV). SVM was also better than naive Bayes in classifying relaxation versus nonrelaxation tweets in accuracy (83.72% vs 78.08%), specificity (77.18% vs 60.00%), and PPV (79.86% vs 70.68%) but slightly lower in sensitivity (90.26% vs 96.15%).

Table 1 also indicates that naive Bayes had better accuracy and sensitivity than SVM in identifying first-hand experience stress and relaxation tweets: 87.58% versus 85.61% (accuracy) and 95.53% versus 90.64% (sensitivity) for stress; 85.64% versus 83.85% (accuracy) and 99.09% versus 95.76% (sensitivity) for relaxation tweets. In contrast, SVM performed better in specificity and PPV in classifying first-hand experience stress and relaxation tweets.

Table 2 shows the terms that had the highest information gain for stress and relaxation classification. Interestingly, we found that most terms characteristic of the stress class were related to the term “stress,” such as “stressed” or “stressin,” In contrast, the terms most characteristic of the relaxation class were “vacation,” “water,” or “beach,” which are related to the topics as categorized in our relaxation schema.

**Table 2:** Top 30 keywords ranked by information gain in stress and relaxation classification in dataset 1.

Stress vs nonstress	First-hand stress vs nonstress	First-hand relaxation vs nonrelaxation	Relaxation vs nonrelaxation
stressed	http	rt	rt
stress	rt	relaxing	relaxing
rt	stressed	relaxin	relaxin
mistress	stressing	sorelaxing	sorelaxing
stressful	stressful	relaxed	relaxed
stressing	mistress	work	time
http	stressingout	night	work
stressingout	sostressed	time	night
cashnewvideo	stressin	day	day
camerondallas	cashnewvideo	shower	cashnewvideo
burdenofstress	school	cashnewvideo	relax
tiger	ly	camerondallas	shower
stressin	stress	finally	camerondallas
sostressed	camerondallas	bath	relaxa
day	day	relax	video
nashgrier	love	listening	finally
distressed	sostressful	beach	bath
school	college	relaxa	home
anxiety	packing	video	vacation
life	life	home	listening
busy	twitter	vacation	beach
learn	tiger	pool	nashgrier
woods	hours	sitting	relaxar
bitch	big	enjoying	pool
hours	nashgrier	watching	enjoying
packing	distressed	rain	rain
twitter	hate	give	long
haha	long	nashgrier	sitting
college	weeks	long	watching
love	figure	bed	nice

### Automatic Classification of Stress and Relaxation Tweets at the City Level (Dataset 2)

Using an SVM algorithm trained on our annotated data (dataset 1), we automatically classified the much larger dataset 2 (cities dataset). We used a 3-step classification process. First, we filtered by the keywords “stress” and “relax.” Second, we applied the stress or relaxation classifier to these filtered data. Third, we used the first-hand classifier to identify first-hand stress and relaxation tweets. In both steps, we used SVM (linear kernel) trained on dataset 1 as the classifier. We used SVM because it had advantages in stress and relaxation classification in comparison with naive Bayes in dataset 1. Table 3 shows the number of tweets after each step.

**Table 3:** Number of tweets remaining after automatic classification.

Cities	Stress rank 2011 (2014)^a^	No. of tweets	No. of tweets containing “relax”	No. of tweets containing “stress”	No. of relaxation tweets	No. of stress tweets	No. of relaxation tweets (first-hand)	No. of stress tweets (first-hand)
Los Angeles	1 (3)	6,627,969	5061	7925	3216	5914	2788	2386
New York	2 (1)	8,229,442	6992	11,789	4412	8245	3766	3278
San Diego	5 (38)	2,908,774	2178	3769	1449	2830	1275	1193
San Francisco	7 (39)	4,372,966	2554	4558	1682	3384	1471	1389

^a^Stress ranking is based on 2011 Forbes [40] and 2014 CNN studies [41]. Statistical tests between cities showed there are differences between cities (*P*<.001), except San Diego and New York (stress: *P*=.18, relaxation: *P*=.02). *P* values of relaxation and stress tweets between San Diego and Los Angeles are .41 and <.001, respectively. Ranks based on stress tweets are New York=San Diego, Los Angeles, and San Francisco.

To evaluate the performance of stress and relaxation classification in dataset 2, we randomly sampled 2 sets of 100 tweets, with each set consisting of 100 tweets containing either the keyword “stress” (set 1) or “relax” (set 2) from a city in dataset 2. We chose New York for evaluation, since New York had the greatest number of tweets. Then 100 tweets from set 1 were manually annotated (conducted by author SD) as stress or nonstress and first-hand experience stress or nonfirst-hand experience stress class. Similarly, 100 tweets from set 2 were also manually annotated as relaxation or nonrelaxation and first-hand relaxation experience or nonfirst-hand experience relaxation class.

**Figure 8:**
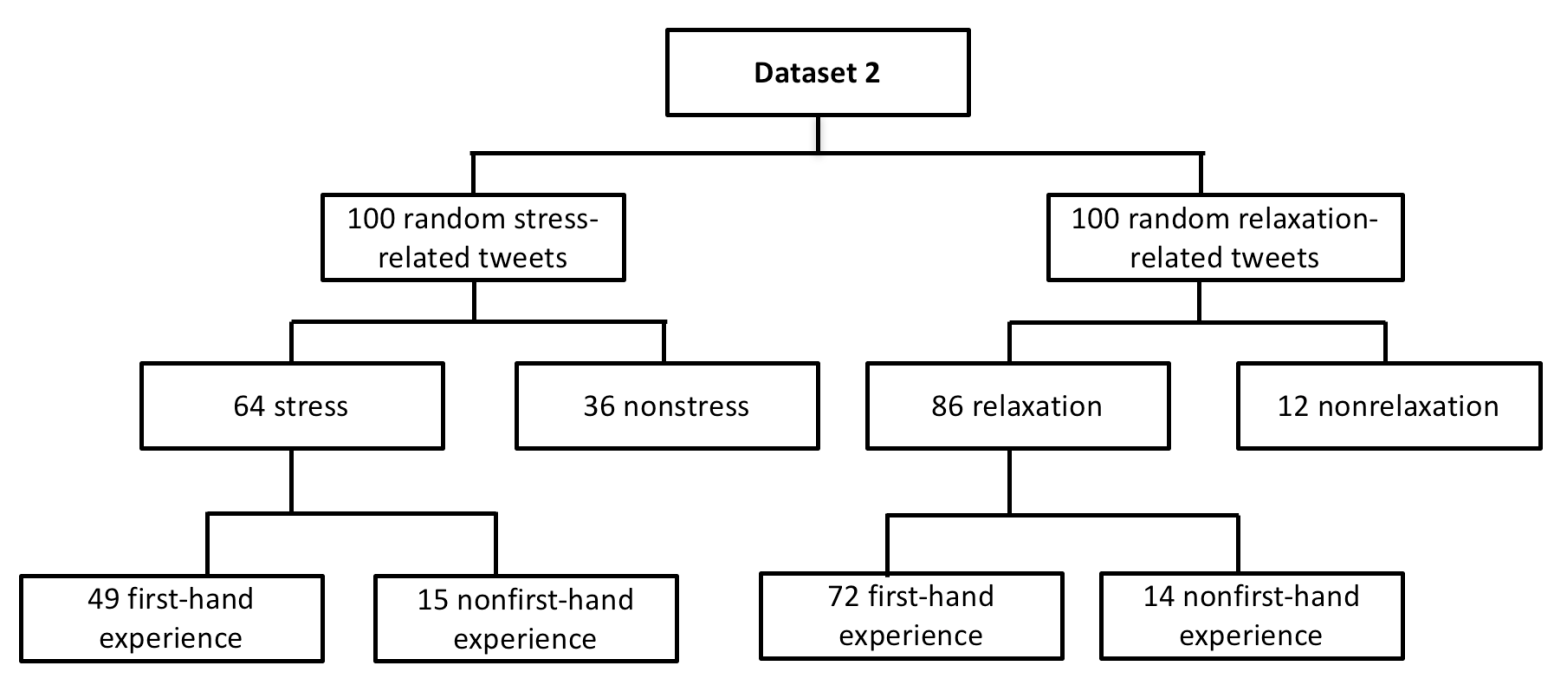
Description of manual annotation of 100 random tweets containing the keywords “stress” and “relax” from dataset 2.

Table 4 shows the results of classification of set 1 and set 2 using the SVM algorithm. It indicated fair accuracy (66.0%-92.0%) and high PPV (84.6%-100.0%); however, it had lower sensitivity in first-hand stress classification (44.0%) and specificity in relaxation classification (57.1%). The results of the SVM algorithm in dataset 2 were different from those in dataset 1, perhaps due to different data distribution. Figure 8 shows the descriptions of manual annotation of 100 random tweets of set 1 and set 2.

**Table 4:** Classification evaluation using a random sample of 200 tweets (100 containing the keyword “stress” and 100 containing the keyword “relax”) from New York in dataset 2.

Classification	SVM (linear kernel)			
	Acc^a^ (%)	Sen^b^ (%)	Spec^c^ (%)	PPV^d^ (%)
Stress vs nonstress	75.0	76.7	70.4	87.5
Relaxation vs nonrelaxation	66.0	67.4	57.1	90.6
First-hand vs nonfirst-hand experience stress	68.0	44.0	92.0	84.6
First-hand vs nonfirst-hand experience relaxation	92.0	87.5	100.0	100.0

^a^Acc: accuracy.

^b^Sen: sensitivity.

^c^Spec: specificity.

^d^PPV: positive predictive value.

**Figure 9:**
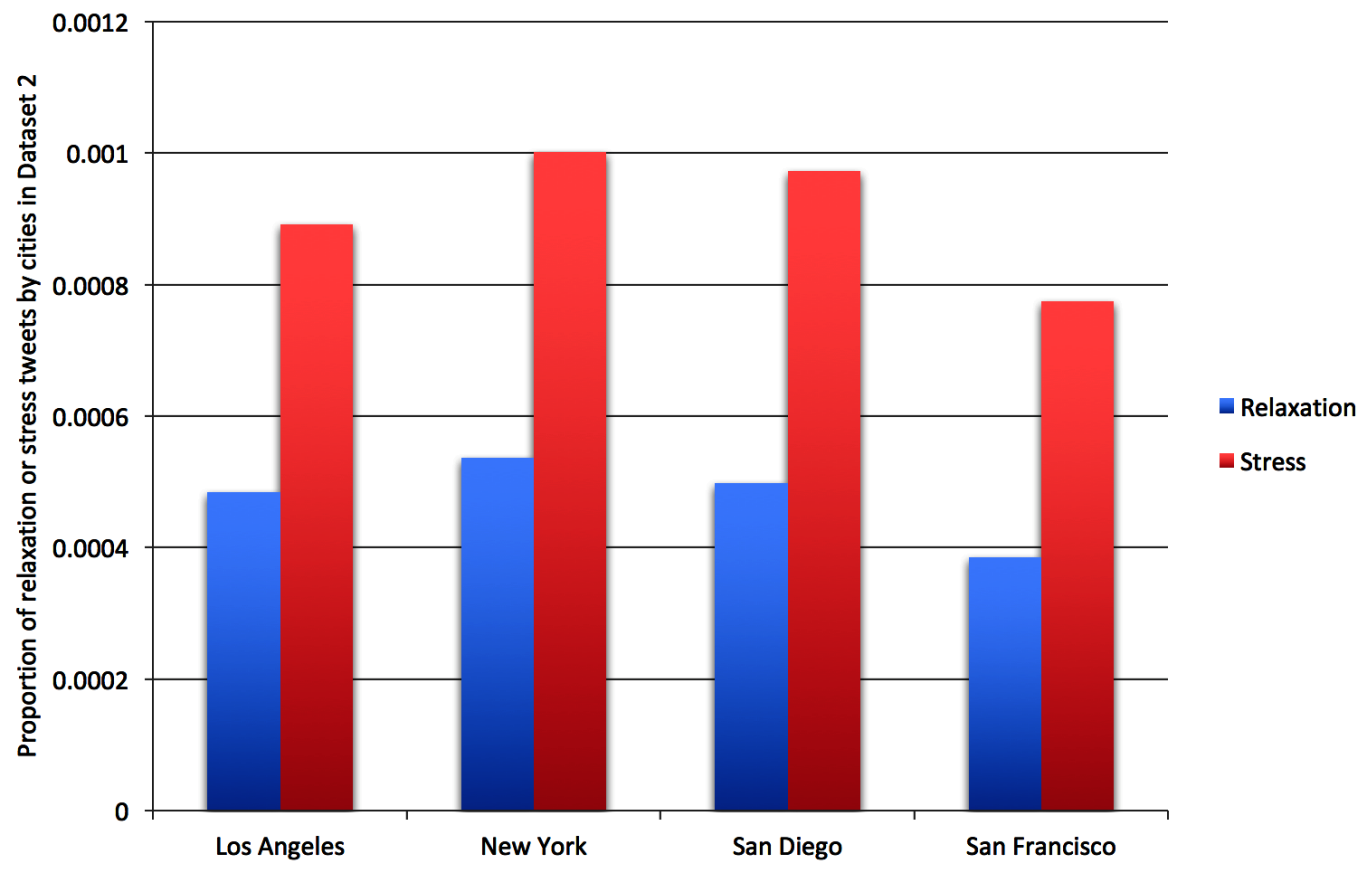
Proportion of relaxation and stress tweets by city in dataset 2.

Figure 9 shows the proportion of stress and relaxation tweets out of all tweets by city in dataset 2. The number of stress tweets was twice that of the number of relaxation tweets, indicating that Twitter users were more likely to tweet about stress than relaxation.

To evaluate theme classification by keyword matching, we randomly sampled 50 classified tweets for each theme from New York. Manual review showed that keyword classification achieved a PPV from 60% to 90% for relaxation tweets and 40% to 80% for stress tweets. Themes that had high PPV in relaxation tweets were alcohol & drugs (94%), entertainment & hobbies (94%), and water (92%). Themes having lower PPV were nature (60%) and food & drink (78%). For stress tweets, themes having high PPV are finances (84%), education (82%), and behavioral (82%), while travel (50%) and temporal (62%) had lower PPV. Multimedia Appendix 3 shows the numbers of classified first-hand stress and relaxation tweets by theme for each city.

First-hand classification results from dataset 2 showed that cities manifested a uniform pattern of stress and relaxation tweets. We found that the singular first-person pronoun “I” was consistently used the most across all cities when expressing stress, found in approximately 4% of all stress tweets, while in relaxation tweets “I” was used less often (ranked 7), at around 2.4%. Multimedia Appendix 4 shows details of the 30 highest-frequency keywords in first-hand experience stress and relaxation tweets for Los Angeles, New York, San Diego, and San Francisco.

We also found that linguistic expressions of negation such as “not,” “but,” and “don’t” or quantifying words such as “much” were among the 30 unigrams most characteristic of stress-related tweets. In addition, users often used emotionally laden swearwords when expressing stress. It is important to note, however, that the affective polarity of certain swearwords can be highly context dependent (“it’s shit” vs “it’s the shit”) [59]. Relaxation tweets, on the other hand, tended to contain words indicating relaxation and time, such as “relax,” “home,” “time,” “day,” and “now.” We found that “home” was among the highest-frequency terms in relaxation tweets, as was “weekend.” Multimedia Appendix 5 depicts tag clouds of stress and relaxation tweets for each city.

### Theme Distributions of Tweets at the City Level (Dataset 2)

**Figure 10:**
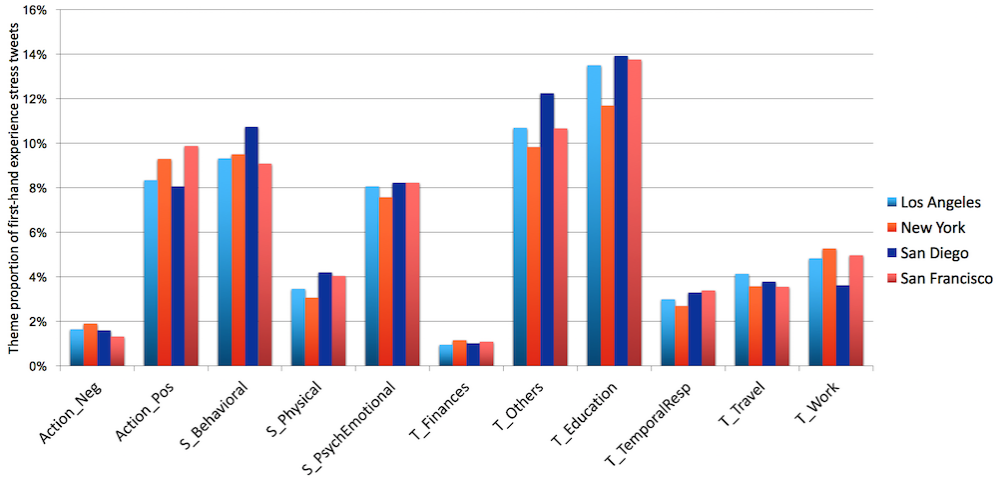
Stress theme distribution by each of the 4 cities in dataset 2. There are no significant differences between cities (*P*>.05). Neg: negative; Pos: positive; S: symptoms; T: topics.

Figure 10 shows the theme distributions of stress tweets among the 4 cities. Education was the highest-frequency topic (12%-14%), followed by work (4%-5%) and travel (4%) (data presented in Multimedia Appendix 3). Interestingly, we found that tweets describing action taken and psychological and emotional symptoms also had relatively high frequencies (8%-10%). This indicates that, besides topic, people often posted about their emotional state and reaction to stress.

**Figure 11:**
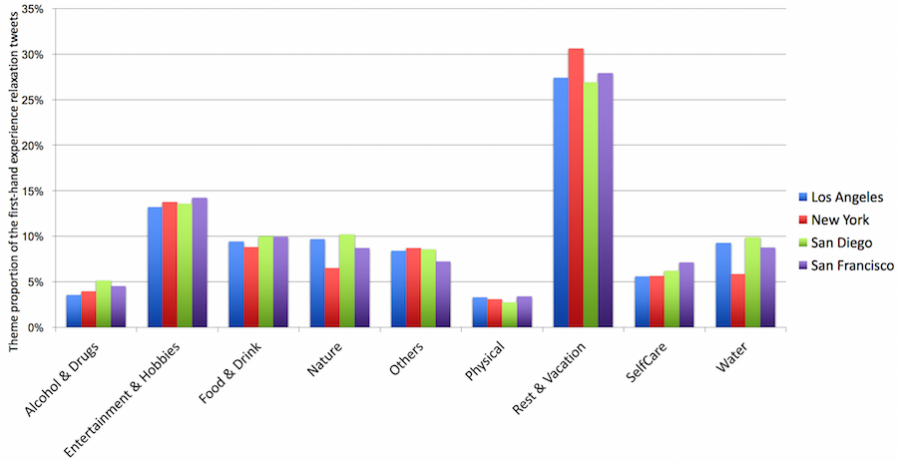
Relaxation theme distribution by each of the 4 cities in dataset 2. There are significant differences between New York and the other cities in the topics of nature and water.

The topic distributions of relaxation tweets were also consistent across cities. Figure 11 shows that rest & vacation was the highest-frequency topic (27%-31%), followed by entertainment & hobbies (13%-14%), food & drink (9%-10%), and nature (9%-10%). Multimedia Appendix 3 shows detailed numbers of stress and relaxation tweets for each city.

Although we did not find statistically significant differences in theme distributions among cities for stress tweets, there were significant differences between New York and the other cities in the topics of nature and water in relaxation tweets. This may indicate the different activities taken for relaxation between the east coast (New York) and the west coast (Los Angeles, San Diego, and San Francisco). We found that high-frequency terms for relaxation tweets in New York included “watching,” while in San Diego “beach” was more common. This intuitively suggests that San Diegans more often relaxed by going to the beach, while New Yorkers relaxed by enjoying indoor (or spectator) entertainment (“watching,” “listening”).

### Correlations Between Tweets Data Analysis and Public Surveys

Compared with 2 public surveys on the most stressful cities in the United States by Forbes [40] in 2011 and CNN [41] in 2014, the proportion of stress tweets found here were different. Both surveys ranked New York and Los Angeles among the most stressful cities in the country, while San Diego and San Francisco were categorized as less stressful. Our city ranking based on the proportion of first-hand experience stress tweets was New York followed by San Diego, Los Angeles, and San Francisco (Table 5 and Figure 9). While we found no significant difference between New York and San Diego, we did find significant differences (*P*<.001) in pairwise comparisons between San Diego, Los Angeles, and San Francisco (Table 5).

**Table 5:** *P* values of pairwise comparisons of the proportion of stress and relaxation tweets between the 4 studied cities.

Cities		Los Angeles	New York	San Francisco
**San Diego**				
	Stress	<.001	.18	<.001
	Relaxation	.41	.02	<.001
**San Francisco**				
	Stress	<.001	<.001	N/A^a^
	Relaxation	<.001	<.001	N/A
**New York**				
	Stress	<.001	N/A	<.001
	Relaxation	<.001	N/A	<.001

^a^N/A: not applicable.

Differences between results found in public stress surveys and our automatic classification of Twitter messages could be due to differences in methodology and population when collecting data. Public surveys collect data using telephones and paper-based reports, while Twitter messages are user generated, are naturalistic, and reflect personal thoughts.

### Stress Relief by Relaxation in Tweets

The distribution of stress topics across cities shows an interesting finding: peoples’ reactions to stress were more positive than negative. Figure 10 shows that, for all cities, 8%-10% of tweets reported positive action taken in response to stress, while only 1%-2% reported negative action (see Multimedia Appendix 3 for details). This suggests that people may react to stress positively, or that people are more likely to publicly report positive rather than negative actions. Examples of positive reaction in stress tweets are rest (“Rest is best when you are stressed”) and exercising (“I’m so stressed, thank god I’m heading to yoga now”).

Relaxation can be considered a stress management activity. Figure 9 shows that the numbers of relaxation tweets were consistently proportional across all cities to those of stress tweets, indicating that Twitter users were consistently more inclined to post about stressful life events or experiences than about relaxing experiences. Examples of stress relief from relaxation tweets are personal contact (“I don’t need anything but a hug...”), exercising (“Went for a run, feel awesome, now time to relax”), shopping (“Last day in #SanDiego Just relaxing, shopping and say bye to friends”), and entertainment (“Relaxing watching a movie:-) :-)”). Figure 7 and Figure 10 also indicate that rest & vacation was the highest-frequency topic within relaxation tweets, followed by entertainment & hobbies, nature, and water. These topics can be considered common activities for stress relief.

## Discussion

### Principal Results

Our research addressed several aspects of the use of Twitter as a medium of expression of stress and relaxation by users. First, we created schema for categorizing stress- and relaxation-related tweets based on previously published psychological guidelines. By categorizing first-hand experience tweets into the primary themes of content topics, symptoms, and actions taken, we gained further insight into the common patterns of expressions of stress.

Second, we analyzed in detail the contents of tweets based on our annotation scheme and found both similarities and differences in the prevalence and characteristics of stress and relaxation tweets across cities on the east and west coasts of the United States. The most frequent topic of stress tweets in our datasets was education, which likely reflects the younger demographic of Twitter users [57,58], but work and travel were also common topics. It is notable that, despite poverty rates, unemployment rates, and cost of living being significant factors in the methodology of CNN’s and Forbes’s stress ranking systems of the most stressful cities, finances were not a major content topic of the stress tweets in any city in our studies. Although this result could be partially attributable to the need for either computer or mobile phone access in order to use Twitter and may cause underrepresentation in lower-income groups, it may also indicate that certain topics, such as personal finances, still remain relatively taboo in social media settings. Regarding positive and negative actions regarding stress, positive actions far outnumbered more destructive behavior. The use of Twitter in itself to discuss feelings of stress and stress management can be seen as a constructive manner of dealing with stress by expressing these feelings and using the support of “followers” and friends. Social media platforms are increasingly being used as support networks in the management of chronic health conditions as varied as cancer, depression, and obesity. A recent systematic review by Patel et al found that the impact of social media use on those experiencing chronic disease was positive in 48% of studies reviewed, neutral in 45%, and harmful in only 7% [60].

Third, our study indicated that words most associated with relaxation strategies (see Table 2) fell into 3 main groups: (1) bathing and personal care (eg, “bath,” “shower”), (2) vacationing (“vacation,” “pool,” “beach”), and (3) watching sports or television (“videos,” “sitting,” “watching”), indicating that relaxation strategies involved purposefully taking time away from work-based activities and daily responsibilities. A further key theme that emerged from a qualitative analysis of the data was the idea of nature—in this case, particularly water (eg, “pool,” “beach,” “rain”)—as being of key importance for relaxation. This result is consistent with recent research demonstrating the link between stress reduction and exposure to the natural environment (eg, [61]).

Finally, we showed that machine learning algorithms could be employed to achieve good accuracy for the automatic classification of stress and relaxation tweets.

### Limitations

This study has several limitations. First, we obtained dataset 2 from the Twitter API’s 1% sample. Second, the annotation scheme we developed, although well suited for our purpose, could benefit from further refinement. For example, we found that many tweets were categorized as topic “other.” Third, it is likely that classification results could be improved given the availability of additional training data, in particular for first-hand experience classification of stress and relaxation tweets. Furthermore, using additional feature sets, such as ngrams, emotions, and negations, could help improve accuracy. Fourth, Twitter reports of stress and relaxation may be influenced by self-presentation issues (eg, stress related to excessive workload can be used as a status indicator in some contexts). Finally, as with all social media-based research, the population studied is unlikely to be a representative sample of the general population.

### Conclusions

This research showed that Twitter can be a useful tool for the analysis of stress and relaxation levels in the community, and has the potential to provide a valuable supplement to social and psychological studies of stress and stress management.

## Appendix

Multimedia Appendix 1 Examples of each category of first-hand experience stress tweets with its themes.

Multimedia Appendix 2 Examples of each category of first-hand experience relaxation tweets with its themes.

Multimedia Appendix 3 Number of classified first-hand stress tweets by theme and first-hand relaxation tweets in each city.

Multimedia Appendix 4 Top 30 highest-frequency keywords in first-hand experience stress and relaxation tweets for Los Angeles, New York, San Diego, and San Francisco.

Multimedia Appendix 5 Tag clouds of stress and relaxation tweets in New York, Los Angeles, San Diego, and San Francisco.

## Abbreviations

API: application programming interface
CDC: Centers for Disease Control and Prevention
PPV: positive predictive value
SVM: support vector machines
