# Supplementary material for: How Do You #relax When You’re #stressed? A Content Analysis and Infodemiology Study of Stress-Related Tweets
Source: JMIR Public Health Surveill. 2017 Jun 13;3(2):e35. doi: 10.2196/publichealth.5939 (PMC5487742; doi:10.2196/publichealth.5939)
Supplement: Multimedia Appendix 1 [file publichealth_v3i2e35_app1.pdf]

Appendix 1. Examples of first-hand experience stress tweets with its themes. Sub-categories of themes are separated by a colon.
Note that all tweets have been paraphrased to preserve user anonymity.

Stress: Symptom: Psychological & Emotional

Examples:

No idea what to do...,, #stressed #worried #lost #frustrated<br>#Broken, #Dejected, #Stressed, #Depressed.
How do I deal with all this. #StressinOut
Fell asleep. Had nightmare. woke up stressed, and somehow my cat bit me. Can things go right for me just once?

Stress: Symptom: Behavioral

Examples:

I need a drink tonight. #sostressed
I'm soooo stressed...I think I might go on an chocolate binge.
im so stressed. started smoking again. hadnt done it in a very long time
Haven't been able to sleep past 9 AM for the past 2 weeks and getting told to do things is just too stressful..

Stress: Symptom: Physical

Examples:

Chest hurts all nighttt #stressing
You get sick and are bedridden for only a few hours but because of the massive workload, you pretty much need to be dead. #stress
Feeling sick and soo stressed from school. Waiting for the weekend to lift my spirits.
Right now, Im shaking and my heart is beating so fast I can't even think! I am broken! Only God can heal me! #stressing

Stress: Symptom: Work

Examples:

This job will kill me! #stress #nosleep
I'm annoyed with my job.. no vacation.. instead i have extra hours and a store without a manager #stressingOut
This is the first time I've cried for a long time, and all because of work. #sostressed
I’m not enjoying work much, to much stress!

Stress: Topic: Education

Examples:

I just wanna finish my homework and go to sleep. This week is going to be horrible #StressingOut

College is #Stressful
Not doing a math course online again #sostressful #whatsgoingon
How can my exam be in less than a month?! #stressing

Stress: Topic: Finances

Examples:

I cannot wait till I get a decent pay check again #stressing
I don't know how I'm gonna afford everything #stressingout
Why do you need to pay for class? #stressingout
I'm really tired of being broke!!!!! #Stressed

Stress: Topic: Relationships & Social

Examples:

Ain't nobody here for me but my kids. #stressingout
My husband going to trial tommorow #Stressingout
Omg my parents are being horrible. i hate them. they put a lot of stress on me. I get blamed for everything.
Freaking out when we don't know where mom is.. #stressingout

Stress: Topic: Responsibilities & Time

Examples:

Known for waiting until the last minute #SoStressful
So many things to do and not enough time to do it in... #StressingOut
Coaches said they want a decision Monday...just stressed now moe
So much to do! thought summers were for chilin #stressingout #needtorelax #ahhhh #cry

Stress: Topic: Travel

Examples:

Don’t like last minute packing. #sostressful
is stressing, I have court today, but not enough gas, =(
why do I always feel so lost down here?? Stressing me out
I don't have enough gas – running out... #stressingout

Stress: Topic: Other

Examples:

When I was a kid, always wanted to be older #sostressful
cleaned my apartment. I've been messy lately I hope things get better. #stressed
Played this week for the 1st time. #stressin
prom is stressing me...and its weeks away!

Stress: Action: Negative

Examples:

I need some nicotine right now... #stressful
I hope my herbal cigarrettes arrive tomorrow, I'm stressed.
I need a drink tonight. #sostressed
Court in the morning – trying to drink away the stress

Stress: Action: Positive

Examples:

I need food, a sleep, and a hug. #stressingout #tired #hungry
Stressful day. #bath #relax
I need to go for a run, getting stressed by this class
smooth jazz is going to get me through the day. #sostressed

Stress: None-specific

Examples:

#stressed!!!
#Stressed about everything!!
Bad Night #SoStressed...
