# Supplementary material for: How Do You #relax When You’re #stressed? A Content Analysis and Infodemiology Study of Stress-Related Tweets
Source: JMIR Public Health Surveill. 2017 Jun 13;3(2):e35. doi: 10.2196/publichealth.5939 (PMC5487742; doi:10.2196/publichealth.5939)
Supplement: Multimedia Appendix 2 [file publichealth_v3i2e35_app2.pdf]

Appendix 2. Examples of first-hand experience relaxation tweets with its themes. Sub-categories of themes are separated by a colon.

Relax: Physical

Examples:

off to spin class to get a good workout. just want to relax:D
Yoga is my new thing #SoRelaxing
My wake up routine is doing 50 crunches, and going for a run. #Relaxing
Awesome workout. I see a relaxing night ahead!

Relax: Water

Examples:

A bubble bath is wonderful after a bad day! #relaxed
Mom’s pool for down time and tanning! #relaxin #poolside
Thunderstrom #SoRelaxing when you’re inside!
Finally some #rain - weather cooled down #relaxed #cool

Relax: Self-Care

Examples:

Take a relaxing night in the hot tub soon.
What an amazing day! Going to have a massage. incredible weather! #luckygirl #sorelaxing http://t.co/EhhTB2Sdeo
Just took a relaxing shower
Pedicures! #relaxing #happydays

Relax: Social

Examples:

Day off . #relaxing
Drinks on a #Monday Lunch coming up #relaxin
A relaxing, stress-free night. can't wait!
just got home from work busy day,glad its over #relax

Relax: Alcohol & Drugs

Examples:

morphine is great! #relaxin
Enjoying a beer, relaxing.. I deserve it!
Off work! I just wanna relax...
All you gotta do is put a #drinkinmyhand and I'm a happy person #relax

Relax: Entertainment & Hobbies

Examples:

I'm gonna do laundry, go in the shower, and relax
#Relaxin doing what they do best
Smoking hookah and watching tV. #SoRelaxing
Best way to enjoy my day off. #Relaxing

Relax: Food & Drink

Examples:

Yesterday: Had a nice #bbq with #friends. We #relaxed, and had #fun.
drinking coffee and generally just relaxing until class begins
raisin bagel, painted nails, city and colour playing and the moon is right outside my window #relaxing
home after a long day, going to have a cup of tea and relax, good night all

Relax: Nature

Examples:

Day off at the beach #relaxin
Sitting here watching the rain #relaxed
I love the rain, helps me relax... I'm so tired
#moodchanger #relaxin #coolin

Relax: Rest & Vacation

Examples:

relaxing and looking at the sunset. Thank goodness for life and being able to breath.
#beach #summer #vacation #sunnyday #smile #loveit #relaxed
It's one of those days. #vacation #relaxed
The conference was amazing! Taking a day off to relax

Relax: Other

Examples:

Been sitting in my bed for an hour #sorelaxing
sometimes I like to just close my eyes...and relax
Bored #selfie #relaxin #lifestyle ...

Relax: None-specific

Examples:

Relaxing
#relaxin
now I wait and relax
just relaxin now too
