# Supplementary material for: How Do You #relax When You’re #stressed? A Content Analysis and Infodemiology Study of Stress-Related Tweets
Source: JMIR Public Health Surveill. 2017 Jun 13;3(2):e35. doi: 10.2196/publichealth.5939 (PMC5487742; doi:10.2196/publichealth.5939)
Supplement: Multimedia Appendix 3 [file publichealth_v3i2e35_app3.pdf]

Appendix 3.

Appendix 3a. Number of classified first-hand stress tweets by theme in each city, the first-hand stress tweets are classified using the SVM classifier.

City	Action_Neg	Action_Pos	S_Behavioral	S_Physical	S_PsychEmotional
Los Angeles	39	199	222	82	192
New York	62	304	311	100	248
San Diego	19	96	128	50	98
San Francisco	18	137	126	56	114

City	T_Finances	T_Others	T_Education	T_Temporal_Resp	T_Travel	T_Work
Los Angeles	22	255	322	71	98	115
New York	37	322	383	88	117	172
San Diego	12	146	166	39	45	43
San Francisco	15	148	191	47	49	69

Appendix 3b. Number of classified first-hand relaxation tweets in each city, the first-hand relaxation tweets are classified using the SVM classifier.

City	AlcoholDrugs	Ent_Hobbies	FoodDrink	Nature
Los Angeles	98	368	262	269
New York	149	518	332	244
San Diego	65	173	127	130
San Francisco	66	209	146	128

City	Others	Physical	Rest_Vaca	SelfCare	Water
Los Angeles	234	91	764	155	258
New York	328	116	1153	212	219
San Diego	109	35	343	79	126
San Francisco	106	50	411	105	129
