# Supplementary material for: How Do You #relax When You’re #stressed? A Content Analysis and Infodemiology Study of Stress-Related Tweets
Source: JMIR Public Health Surveill. 2017 Jun 13;3(2):e35. doi: 10.2196/publichealth.5939 (PMC5487742; doi:10.2196/publichealth.5939)
Supplement: Multimedia Appendix 4 [file publichealth_v3i2e35_app4.pdf]

Appendix 4

Appendix 4a. Top 30 highest frequency keywords in first-hand experience stress tweets for Los Angeles, New York, San Diego, and San Francisco.

Stress category											
Los Angeles			New York			San Diego			San Francisco		
i	2692	4%	i	3604	4.10%	i	1216	3.90%	i	1626	4.10%
stress	2191	3.30%	stress	2873	3.20%	stress	968	3.10%	stress	1198	3%
to	1739	2.60%	to	2332	2.60%	to	840	2.70%	to	1069	2.70%
the	1473	2.20%	the	2032	2.30%	the	696	2.20%	the	834	2.10%
out	1330	2%	stressed	2020	2.30%	stressed	662	2.10%	stressed	804	2%
stressed	1330	2%	a	1687	1.90%	out	623	2%	out	755	1.90%
my	1228	1.80%	my	1621	1.80%	is	611	2%	my	746	1.90%
and	1185	1.80%	is	1576	1.80%	my	595	1.90%	and	741	1.90%
is	1144	1.70%	out	1447	1.60%	so	581	1.90%	is	678	1.70%
a	1136	1.70%	and	1444	1.60%	and	556	1.80%	a	657	1.70%
so	1072	1.60%	so	1381	1.60%	me	505	1.60%	so	632	1.60%
me	1031	1.50%	me	1243	1.40%	a	498	1.60%	me	607	1.50%
i'm	904	1.40%	this	1129	1.30%	i'm	459	1.50%	i'm	541	1.40%
stressful	870	1.30%	stressful	1125	1.30%	stressful	435	1.40%	stressful	497	1.30%
this	757	1.10%	i'm	1095	1.20%	this	384	1.20%	this	477	1.20%
stressing	655	1%	of	833	0.90%	of	319	1%	of	396	1%
of	640	1%	stressing	811	0.90%	stressing	313	1%	stressing	385	1%
it	585	0.90%	for	752	0.80%	for	284	0.90%	it	357	0.90%
for	543	0.80%	it	749	0.80%	it	263	0.80%	for	325	0.80%
be	499	0.80%	be	738	0.80%	be	244	0.80%	just	299	0.80%
just	490	0.70%	that	661	0.70%	just	239	0.80%	be	295	0.70%
that	459	0.70%	in	638	0.70%	about	224	0.70%	about	280	0.70%
you	452	0.70%	just	630	0.70%	that	216	0.70%	in	272	0.70%
about	444	0.70%	you	551	0.60%	much	206	0.70%	that	267	0.70%
in	440	0.70%	about	535	0.60%	have	196	0.60%	you	249	0.60%
have	374	0.60%	have	497	0.60%	in	192	0.60%	have	220	0.60%
much	374	0.60%	not	494	0.60%	you	190	0.60%	not	214	0.50%
all	362	0.50%	all	490	0.60%	all	185	0.60%	all	213	0.50%
not	350	0.50%	much	485	0.50%	not	171	0.50%	much	212	0.50%
			don't	470	0.50%	but	161	0.50%	but	205	0.50%
over	340	0.50%									

Appendix 4b. Top 30 highest frequency keywords in first-hand experience relaxation tweets for Los Angeles, New York, San Diego, and San Francisco.

Relaxation category											
Los Angeles			New York			San Diego			San Francisco		
relax	1571	3.80%	relax	2296	4.10%	relax	706	3.80%	relax	811	3.60%
to	1397	3.40%	to	1794	3.20%	to	620	3.30%	to	768	3.40%
and	1266	3.10%	and	1692	3%	and	595	3.20%	and	758	3.40%
a	1152	2.80%	relaxing	1463	2.60%	relaxing	524	2.80%	a	585	2.60%
relaxing	1138	2.80%	the	1346	2.40%	the	489	2.60%	relaxing	585	2.60%
the	1057	2.60%	a	1335	2.40%	a	466	2.50%	i	547	2.40%
i	957	2.30%	i	1317	2.40%	i	405	2.20%	the	526	2.40%
t	653	1.60%	my	817	1.50%	t	371	2%	my	318	1.40%
co	650	1.60%	just	714	1.30%	co	366	2%	just	287	1.30%
http	644	1.60%	t	685	1.20%	http	361	1.90%	t	281	1.30%
just	565	1.40%	co	680	1.20%	my	248	1.30%	co	277	1.20%
my	537	1.30%	http	672	1.20%	just	247	1.30%	http	275	1.20%
so	418	1%	so	538	1%	so	185	1%	in	228	1%
time	412	1%	with	537	1%	in	184	1%	so	214	1%
in	411	1%	in	535	1%	time	183	1%	time	214	1%
of	389	0.90%	is	523	0.90%	is	171	0.90%	is	209	0.90%
with	377	0.90%	of	505	0.90%	at	161	0.90%	for	200	0.90%
is	356	0.90%	time	467	0.80%	day	157	0.80%	of	198	0.90%
day	341	0.80%	day	453	0.80%	of	156	0.80%	day	190	0.90%
for	329	0.80%	for	432	0.80%	for	151	0.80%	with	182	0.80%
on	308	0.80%	on	427	0.80%	with	146	0.80%	on	166	0.70%
at	304	0.70%	home	424	0.80%	on	142	0.80%	at	159	0.70%
it	250	0.60%	at	360	0.60%	it	115	0.60%	now	150	0.70%
it's	242	0.60%	work	355	0.60%	home	102	0.50%	it	147	0.70%
now	232	0.60%	now	331	0.60%	it's	99	0.50%	home	127	0.60%
you	219	0.50%	i'm	323	0.60%	me	97	0.50%	some	120	0.50%
this	218	0.50%	like	311	0.60%	this	94	0.50%	i'm	116	0.50%
home	207	0.50%	this	310	0.60%	you	94	0.50%	me	114	0.50%
me	206	0.50%	me	292	0.50%	now	93	0.50%	it's	108	0.50%
i'm	194	0.50%	back	285	0.50%	have	88	0.50%	you	104	0.50%
