# Supplementary figures and images for: How Do You #relax When You’re #stressed? A Content Analysis and Infodemiology Study of Stress-Related Tweets
Source: JMIR Public Health Surveill. 2017 Jun 13;3(2):e35. doi: 10.2196/publichealth.5939 (PMC5487742; doi:10.2196/publichealth.5939)

## Appendix 5.

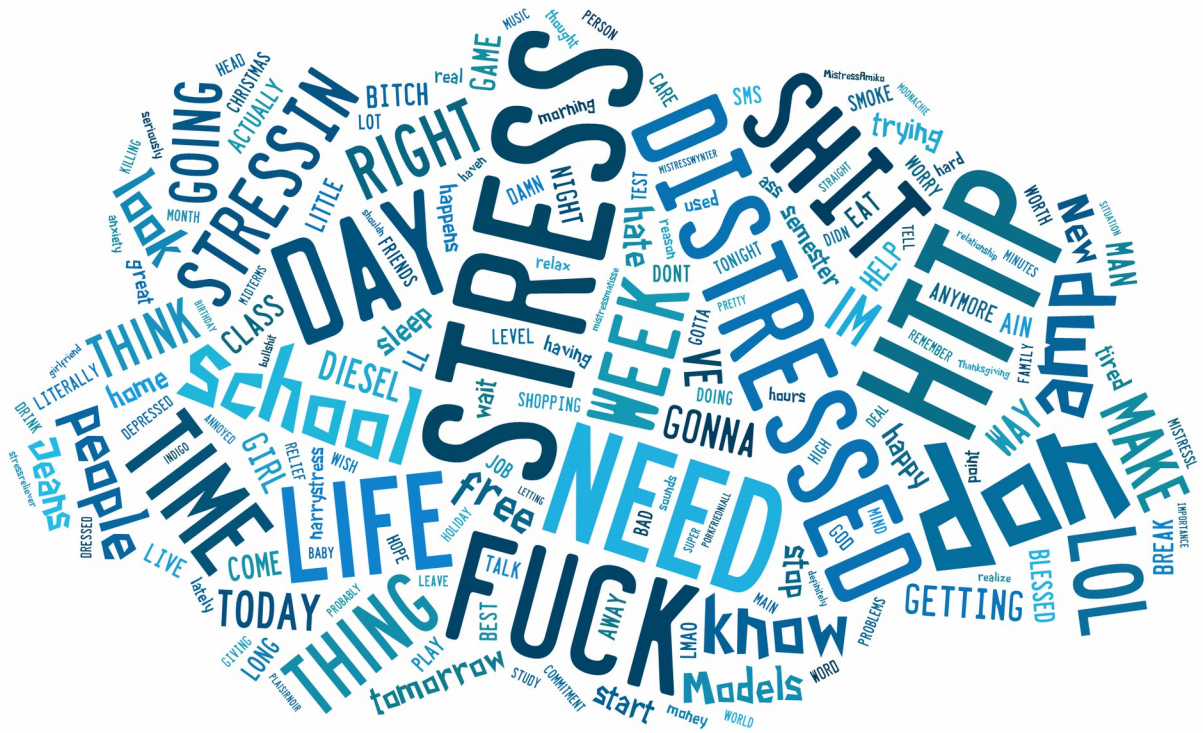

**Figure 12.** Tag clouds of stress tweets in New York.

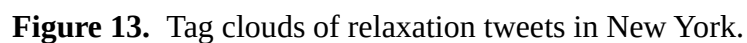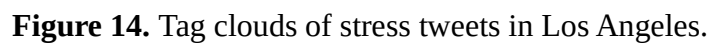

Supplement: Multimedia Appendix 5 [file publichealth_v3i2e35_app5.pdf]
